# Dermatologic Vasculature Diseases as a Risk Factor of Subconjunctival Hemorrhage

**DOI:** 10.3390/ijerph16162865

**Published:** 2019-08-10

**Authors:** Chia-Yi Lee, Hung-Chi Chen, Jing-Yang Huang, Chi-Chin Sun, Chao-Bin Yeh, Hung-Yu Lin, Shun-Fa Yang

**Affiliations:** 1 Department of Ophthalmology, Show Chwan Memorial Hospital, Changhua 500, Taiwan; 2 Department of Optometry, College of Medicine and Life Science, Chung Hwa University of Medical Technology, Tainan 717, Taiwan; 3 Department of Ophthalmology, Chang Gung Memorial Hospital, Linkou 333, Taiwan; 4 Department of Medicine, Chang Gung University College of Medicine, Taoyuan 333, Taiwan; 5 Center for Tissue Engineering, Chang Gung Memorial Hospital, Linkou 333, Taiwan; 6 Department of Medical Research, Chung Shan Medical University Hospital, Taichung 402, Taiwan; 7 Department of Ophthalmology, Chang Gung Memorial Hospital, Keelung 204, Taiwan; 8 Department of Chinese Medicine, Chang Gung University, Taoyuan 333, Taiwan; 9 Department of Emergency Medicine, Chung Shan Medical University Hospital, Taichung 402, Taiwan; 10 Institute of Medicine, Chung Shan Medical University, Taichung 402, Taiwan; 11 Department of Optometry, Chung Shan Medical University, Taichung 402, Taiwan; 12 Department of Exercise and Health Promotion, Chung Chou University of Science and Technology, Changhua 500, Taiwan

**Keywords:** subconjunctival hemorrhage, vascular, hemangioma, teleangiectasias, dermatological

## Abstract

To evaluate the relationship between subconjunctival hemorrhage (SCH) and dermatologic vasculature diseases (DVDs) via the national health insurance research database (NHIRD) of Taiwan. This retrospective cohort study used data from the NHIRD for the 2009 to 2013 period. Patients diagnosed with DVDs were enrolled in the study group, and a propensity score-matching population was selected as the control group after exclusion. The main outcome was set as the development of SCH in both groups. Multivariable Cox regression analysis and survival analysis were performed to estimate the adjusted hazard ratio (aHR) and cumulative probability of SCH. A total number of 3426 patients were enrolled and split equally into the study and the control groups. There was no prominent difference between the age, gender, urbanization, income level, systemic co-morbidities, and ocular diseases between the two groups after matching. During the whole study period, 131 patients in the study group and 98 patients in the control group developed SCH with a significant higher aHR of 2.69 in the study group (*p* < 0.05). In the survival analysis, the study group also demonstrated a higher cumulative probability of developing SCH than the control group throughout the study period (*p* = 0.02). In conclusion, the presence of DVDs may be a risk factor for the development of SCH.

## 1. Introduction

Subconjunctival hemorrhage (SCH) is a benign ocular disorder of which the annual incidence is nearly one percent in the eastern Asia population [1]. The clinical feature of SCH is an asymptomatic circumscribed redness of hemorrhage under the conjunctival surface with rupture of the conjunctival capillary, in which the lesion can resolve spontaneously two weeks after the occurrence [2]. The idiopathic form of SCH tends to occur in the elderly while other ocular disorders including conjunctivitis, eye trauma, surgery-induced lesions, conjunctivochalasis, and contact lens usage are potential risk factors [3,4,5,6].

Systemically, hypertension has been considered to be a cause of SCH with a two-fold increase in the risk than the control [7]. Diabetes mellitus has a similar trend. [5] In addition, SCH has been observed in patients with coagulopathies, including uremic syndrome, dengue hemorrhagic fever, leptospirosis, and use of anticoagulation agents [8,9,10,11]. Also, diseases that interrupt vascular structures like Steve-Johnson syndrome and hemochromatosis have been reported to present as SCH [12,13]. Given that the common risk factors of SCH are mainly related to the vascular and hematological abnormality, we wonder if there is any correlation between SCH and other diseases of vascular-origin diseases.

Likewise, dermatologic vasculature diseases (DVDs) including Kaposi’s sarcoma, hemangiomas, pyogenic granuloma, teleangiectasias, and superficial capillary abnormalities are not uncommon in the skin with enriched microvascular structures [14,15,16,17]. However, evidence that links SCH and those DVDs is still missing, although SCH does occur in ocular disorders of vascular-origin, such as conjucntivallymphangioma and ocular cavernous hemangioma [18,19].

Herein, we aimed to evaluate the relationship between SCH and DVDs using the national health insurance research database (NHIRD) of Taiwan. In addition to DVDs, other systemic co-morbidities were also examined in the multivariate analysis model to investigate whether DVDs is an independent risk factor for SCH which develops in a majority of the population.

## 2. Materials and Methods

### 2.1. Data Source

This retrospective population-based cohort study was approved by the National Health Insurance Administration and the Institutional Review Board of Chung Shan Medical University (Registration Number: CSMUH CS2-15061). The NHIRD is maintained by the National Health Research Institute and contains insurance claim data. The National Health Insurance program covered more than 99% of the entire population of Taiwan in 2010. The longitudinal health insurance database (LHID) 2010 includes the data of one million individuals who were randomly sampled from the NHIRD and registered at the end of 2010. This study was conducted using data collected from the LHID for the period from 1 January 2009, to 31 December 2013. The diagnostic codes were recorded according to the International Classification of Diseases, Ninth Revision (ICD-9). Medications prescribed to patients and their demographics (e.g., socioeconomic status and residential area) can also be found in the NHIRD.

### 2.2. Patient Selection

Patients diagnosed with the following DVDs were enrolled in the study group of the current study: (1) Kaposi’s sarcoma (ICD-9 code: 176.x), (2) hemangiomas (ICD-9 code: 228.0x), (3) pyogenic granuloma (ICD-9 code: 686.1), and (4) teleangiectasias and superficial capillary disorders (ICD-9 code: 448.9). To further enhance the diagnostic accuracy in the study group, only patients received the above ICD-9 code from a dermatologist (department code: 11) were included to ensure the vascular lesions exactly as they came from the skin. In addition, patients were excluded if any of the criteria occurred to erase the confounding factors as possible: (1) received any ocular surgery in the study period, (2) the application of Aspirin (medicine code: A023534100, A024465100, A036599100, A0429341G0, A044016100, A0440161G0, AC37344100, AC373441G0), Clopidogrel (medicine code: AA48649100, AA50126100, AC55026100, AC57819100, BC24863100) and Warfarin (medicine code: B020516100, B023426100) throughout the study period, (3) the presence of ocular trauma (ICD-9 codes: 871.x, 918.x, 921.x) (4) the diagnosis of SCH before the index date which set as the first date of DVDs diagnosis, and (5) age younger than 20 years old or older 100 years. In addition, the individuals in the study group were matched to a non-DVDs individual that serves as the control group, and DVDs patient who could not be matched with a non-DVDs patient were excluded.

### 2.3. Main Outcome Measurement

The primary outcome in the current study was the development of SCH which with the ICD-9 code of 372.72 and diagnosed by an ophthalmologist (department code: 10) after the index date. Since the acute hemorrhagic conjunctivitis, an inflammatory disease with ocular surface hemorrhage, has a different ICD-9 code in practice (ICD-9 code: 077.4 and 372.30), the possibility to misdiagnose acute hemorrhagic conjunctivitis as SCH by ophthalmologist is minimal. Moreover, we also considered the effect of demographic conditions (i.e., age, gender, urbanization and income level) and the following Charlson comorbidity index (CCI) to standardize the health status in the study population: hypertension (ICD-9 codes: 401–405), diabetes mellitus (DM) (ICD-9 codes: 250.x), acute ischemic heart disease (ICD-9 codes: 410–413), congestive heart failure (ICD-9 codes: 398.91, 402.01, 402.11, 402.91, 404.01, 404.03, 404.11, 404.13, 404.91, 404.93, 425.4–425.9, 428.x), peripheral vascular disease (ICD-9 codes: 093.0, 437.3, 440.x, 441.x, 443.1–443.9, 47.1, 557.1, 557.9, V43.4), cerebrovascular disease (ICD-9 codes: 362.34, 430.x–438.x), dementia (ICD-9 codes: 290.x, 294.1, 331.2), chronic pulmonary disease (ICD-9 codes: 416.8, 416.9, 490.x–505.x, 506.4, 508.1, 508.8), rheumatic disease (ICD-9 codes: 446.5, 710.0–710.4, 714.0–714.2, 714.8, 725.x), peptic ulcer disease (ICD-9 codes: 531.x–534.x), liver disease (ICD-9 codes: 070.22, 070.23, 070.32, 070.33, 070.44, 070.54, 070.6, 070.9, 456.0–456.2, 570.x, 571.x, 572.2–572.8, 573.3, 573.4, 573.8, 573.9, V42.7), hemiplegia or paraplegia (ICD-9 codes: 334.1, 342.x, 343.x, 344.0–344.6, 344.9), renal disease (ICD-9 codes: 403.01, 403.11, 403.91, 404.02, 404.03, 404.12, 404.13, 404.92, 404.93, 582.x, 583.0–583.7, 585.x, 586.x, 588.0, V42.0, V45.1, V56.x), malignancy including lymphoma and leukemia, but excluding malignant neoplasm of the skin (ICD-9 codes: 140.x–172.x, 174.x–195.8, 200.x–208.x, 238.6) and coagulation defects (ICD-9-CM codes 286.x). About the ocular co-morbidities, corneal diseases (ICD-9 codes 370.0x, 370.2x, 370.3x, 370.4x, 370.5x, 370.6x, 371.0x, 371.23, 371.4x, 371.6x), cataract (ICD-9 codes 366.10–366.19, 366.8, 366.9), glaucoma (ICD-9 codes 365.x), age-related macular degeneration (AMD) (ICD-9 codes 362.50, 362.51, 362.52), blepharitis (ICD-9 codes 373.0), chronic conjunctivitis including vernal and allergic type (ICD-9 codes 372.1), and noninfectious dermatitis of eyelid including eczematous and allergic type (ICD-9 codes 373.31, 373.32) were considered in the analysis model. We longitudinally traced the data from the index date until the date of SCH diagnosis, withdrawal from the national health insurance program, or 31 December 2013.

### 2.4. Statistical Analysis

SAS version 9.4 was employed for the analysis. We used the propensity score matching to deal with the potential confounders. The propensity score (predicted probability of DVDs exposure) was estimated for each individual by logistic regression, and the factors included birth year (a difference of age within one year was regarded as the same age), gender, urbanization, income, diseases in the CCI presented as different grade, corneal diseases, cataract, glaucoma, AMD, blepharitis, chronic conjunctivitis and noninfectious dermatitis of eyelid. To standardize the health status more precisely, the percentage of hypertension, DM and renal disease between the two groups were considered separately. The paired DVDs and control individuals were 1:1 matched when the difference of propensity score was nearest between exposure and control. An absolutely standard difference (ASD) was employed to show the differences in the demographic data (age, gender, and income level), and co-morbidities status between the study and control groups after the propensity score matching, the ASD less than 0.1 means the item was balanced between two groups. Then the incidence rate ratio and corresponding 95% confidence intervals (CI) were calculated by Poisson regression. Cox proportional hazard regression that was conducted by including all the demographic data, prominent ocular diseases and systemic co-morbidities mentioned above in the multivariable model that was adopted to compute adjusted hazard ratios (aHR). We plotted Kaplan–Meier curves to indicate the cumulative incidence proportion of SCH between the study and control groups with an interval of four years after the DVDs diagnosis and used the log rank test to determine the significant difference between the survival curves. Since most patients in the NHIRD are Han Taiwanese, race was not considered as a covariate. Results with *p* < 0.05 were regarded as statistically significant and a *p* value of less than 0.01 were depicted as *p* < 0.01.

## 3. Results

**Figure 1:**
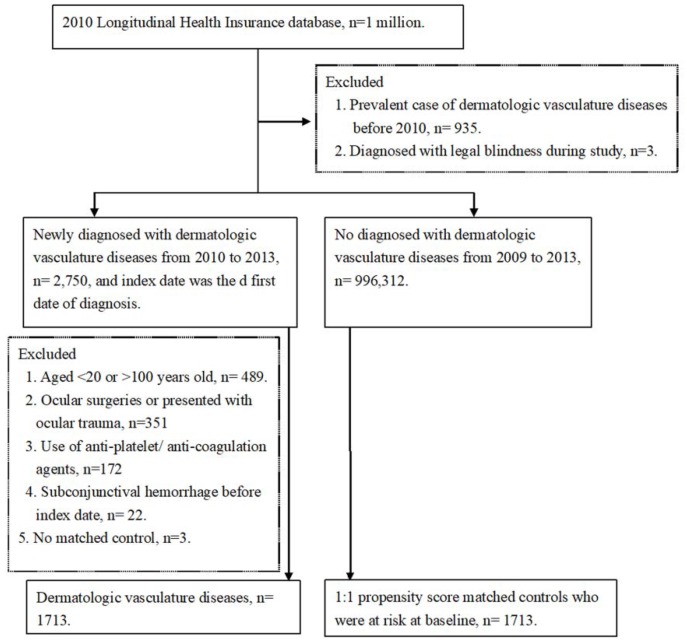
Flowchart of patient enrollment with and without dermatologic vasculature disease.

The flowchart of patient selection and exclusion was shown in Figure 1. After the inclusion and matching, a total number of 3426 patients were selected and split equally into the study and the control groups. There was no prominent difference between the age, gender, urbanization, income level, CCI, and ocular diseases between the study and the control groups after matching, and the details of basic characters and the standardized difference between groups are shown in Table 1.

During the whole study period, 131 patients in the study group and 98 patients in the control group developed SCH. The incidence rate (per 1000 person months) was 3.17 in the study group which was higher than that in the control group (2.35). After adjustment in the multivariate model, the study group yielded a significant higher aHR of 2.69 compared to the control group (*p* < 0.05). Still, no other factors, including demographic data, socioeconomic level, prominent ocular diseases, and systemic co-morbidities, were correlated to the occurrence of SCH. The risk of developing SCH in both groups was shown in Table 2.

**Figure 2:**
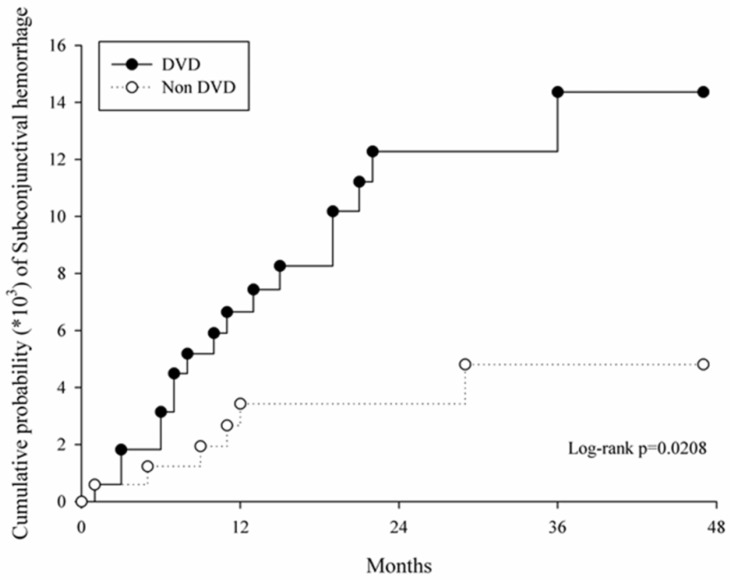
Curves with cumulative proportion of subconjunctival hemorrhage in the propensity score matched study and control groups.

In the survival analysis, the study group demonstrated a higher cumulative probability of developing SCH than the control group throughout the study period (*p* = 0.02, Figure 2). The sensitivity analysis revealed no significant association between the occurrence of SCH and demographic data in the study group (Table 3).

## 4. Discussion

Briefly, we demonstrated the significant correlation between SCH and certain types of DVDs including Kaposi’s sarcoma, hemangiomas, pyogenic granuloma, teleangiectasias, and superficial capillary disorders in the current study. Moreover, the cumulative probability of SCH was significantly higher in the study group in the total four years period.

Various systemic vascular disorders are associated with the occurrence of SCH including hypertension, DM, venous congestion, hematological dyscrasias and menstruation [2,5]. About local vascular lesions in eye, lymphangioma, cavernous hemangioma, conjunctival lymphangiectasia, and Kaposi’s sarcoma are correlated with SCH [2,18,19], which could also develop as dermatological lesions [16,17]. Concerning the mechanism on the vasculature, weaken and fragile vessel is the main cause of SCH [2], while dysfunction and abnormality of the small vessel also occurred in DVDs [14,15,16,17,20,21,22]. Moreover, the process of angiogenesis would be altered in patients with DVDs [23,24], which would influence the vasculature in the whole body and may lead to the development of SCH. The incidence rate ratio of SCH in the study group was significantly higher than that in the control group, which supported this conception.

About the relationship between SCH and DVDs, the current study demonstrated the significant relationship between the two disorders (aHR = 2.69), which the risk of developing SCH elevated by time (*p* = 0.02, log-rank test). To our knowledge, this is preliminary research that tries to illustrate the DVDs as a risk factor for SCH. The etiologies of DVDs including in the current study are different. Kaposi’s sarcoma is viral origin while teleangiectasias may result from scleroderma [17,21]. Nevertheless, the vascular abnormality discussed in the earlier section implies that all the DVDs enrolled in the current study may lead to the presence of SCH. Moreover, the majority of known risk factors of SCH including hypertension, DM, coagulation defect, and ocular disorders were matched and adjusted in the analysis and individuals that received ocular surgery, experienced ocular trauma, and received anticoagulation agent therapy were excluded. The above selections implied that the difference of SCH development between the two groups were due to the exposure of DVDs most likely. Concerning the percentage of SCH occurrence, the study group in the current study showed a 7.65% which higher than the generally 6.77% in the same population from a previous study [1]. Also, in the study written by Kaimbo et al. that survey the occurrence of SCH with a 5 years period, the percentage was 0.80 which lower than the current study [25]. The above evidence suggested that DVDs may be an independent risk factor for SCH.

For other factors included in the current study, no significant correlation was observed to the development of SCH. Although several known risk factors demonstrated in the previous study were also enrolled in the analysis [2,5], the study group, and the control group were propensity score-matched so the distribution of systemic disease would be similar and erase the effect of potential risk factors. Interestingly, the age did not enhance the development of SCH in the sensitivity analysis which proven as a risk factor of SCH in previous research [5]. The possible explanations are that the presence of DVDs overwhelms the influence of age, and the effect of age on SCH results from hypertension that increases with age [26].

There are some limitations to the current study. First, the severity of diseases, including the SCH, DVDs, and the co-morbidities used to standardize the health status like hypertension and DM, cannot be evaluated due to the absence of medical chart review which would lead to less accuracy on outcome evaluation and health status standardization. Second, the contact lens induced SCH cannot be investigated since the contact lens is self-paid in Taiwan mostly, and we can only match the corneal diseases in both groups to standardize the ocular surface status as possible. Also, the DVDs considered in the current study have different etiologies: the pyrogenic granuloma is a vascular proliferation that commonly occurs after trauma while Kaposi’s sarcoma owns a viral etiology [15,17]. The hemangiomas showed neoplastic origin, while teleangiectasias is associated with hereditary factor [14,16]. The different etiologies among DVDs included in the study group would be a major defect in the interpretation of the results which the exact pathophysiology for the causal relationship between those DVDs and following SCH becomes unclear. Furthermore, conjunctivochalasis cannot be excluded due to the rare use of corresponding diagnostic code (ICD-9 code: 37.281) in clinical practice, and no patient with this diagnosis was found in the current study. Nevertheless, the conjunctivochalasisis related to older age [27], so the influence may be limited since we matched the age via propensity scores between both groups.

## 5. Conclusions

In conclusion, the presence of DVDs would be a risk factor for the development of SCH. Further study should be conducted to evaluate the relationship between each DVDs or other systemic vasculature abnormalities and SCH.

## Figures and Tables

**Table 1:** Basic characteristic at baseline in the study group and the matched control group.

	DVD *n* = 1713	Control *n* = 1713	ASD
Age at baseline, Mean ± SD	46.96 ± 14.45	46.27 ± 14.34	0.046
<40	571(33.33%)	606(35.38%)	
40–59	821(47.93%)	805(46.99%)	
≥60	321(18.74%)	302(17.63%)	
Sex			0.043
Female	952(55.58%)	978(57.09%)	
Male	761(44.42%)	735(42.91%)	
Urbanization			0.062
Urban	1133(66.14%)	1179(68.83%)	
Sub-urban	448(26.15%)	422(24.64%)	
Rural	132(7.71%)	112(6.54%)	
Low income	18(1.05%)	17(0.99%)	0.006
CCIs			0.024
0	789(46.06%)	801(46.76%)	
1–2	660(38.53%)	651(38.00%)	
≥3	264(15.41%)	261(15.24%)	
Co-morbidities			
Corneal disease	81(4.73%)	74(4.32%)	0.020
Cataract	93(5.43%)	82(4.79%)	0.029
Glaucoma	31(1.81%)	24(1.40%)	0.033
AMD	8(0.47%)	2(0.12%)	0.065
Blepharitis	29(1.69%)	32(1.87%)	0.013
Chronic conjunctivitis	314(18.33%)	270(15.76%)	0.068
Noninfectious dermatitis of eyelid	3(0.18%)	2(0.12%)	0.015
Hypertension	304(17.75%)	295(17.22%)	0.014
DM	145(8.46%)	142(8.29%)	0.006
Renal disease	49(2.86%)	53(3.09%)	0.014

DVD = dermatologic vasculature disease, SD = standard deviation, CCI = Charlson comorbidity index, AMD = age-related macular degeneration, DM = diabetes mellitus, ASD = absolutely standard difference.

**Table 2:** Incidence risk of subconjunctival hemorrhage between the study group and the matched control group.

Incidence	DVD (*n* = 1713)	Control (*n* = 1713)
Follow up person months	41,339	41,659
Event of Subconjunctival hemorrhage	131	98
Incidence rate * (95% CI)	3.17 (2.67–3.76)	2.35 (1.93–2.87)
Crude HR (95% CI)	2.85 (1.13–7.24)	Reference
Adjusted HR (95% CI)	2.69 (1.05–6.84)	Reference

* per 1000 person months, DVD = dermatologic vasculature disease, CI = confidence interval, HR = hazard ratio.

**Table 3:** Sensitivity analysis of the risk of subconjunctival hemorrhage in the study group.

Subgroups	aHR	95% CI	*p* Value
Sub-group-sex			
Female	2.97	0.94–9.36	0.0628
Male	3.89	0.60–25.11	0.1540
Sub-group-age			
<40	Cannot estimated	-	-
40–59	2.31	0.72–7.43	0.1595
≥60	2.17	0.4–11.91	0.3730

aHR = adjusted hazard ratio, CI = confidence interval.
